# Palliative care for cancer patients in veterinary medicine

**DOI:** 10.17221/76/2022-VETMED

**Published:** 2023-01-15

**Authors:** Claudia Sampaio Fonseca Repetti, Janaina Reato Rueda, Camila Dias Porto, Rodrigo Prevedello Franco, Raul Jose Silva Girio, Fabio Fernando Ribeiro Manhoso, Isabela Bazzo da Costa

**Affiliations:** Department of Veterinary Science, University of Marília, Marília/SP, Brazil

**Keywords:** animal care, dog, euthanasia, neoplasms, quality of life, well-being

## Abstract

Neoplasms account for approximately half of the deaths of dogs over ten years of age. This finding, associated with the fact that canine cancer patients will often suffer from the consequences of the systemic spread of the tumour and paraneoplastic syndromes, shows the importance of understanding palliative care by veterinarians and owners. In view of this, this review aims to address palliative care that can be used in veterinary medicine to promote the patient’s well-being and quality of life.

## INTRODUCTION

Buddhists often say that “pain is inevitable, but suffering is optional”. Given this statement, it can be inferred that our obligation in veterinary medicine is to recognise, alleviate, and prevent the pain and suffering of the animals entrusted to our care. In view of this, the veterinarian often needs to be aware of the guidelines for pain control and maintenance of the patient’s quality of life (Bishop et al. 2016).

Companion animals, in general, have now won a place in the bedroom and on the sofa while previously occupying the backyard and kennel of our homes. The evolution of pets as family members, associated with advances in preventive, interventional, and diagnostic medicine, has resulted in a longer life expectancy for dogs and cats. The direct consequence of this increased longevity is the emergence of age-related diseases including neoplasms in various systems (Downing 2020).

Cancer corresponds to a group of diseases that stand out for cell growth and proliferation that is beyond the control of the organism (Dibernardi 2020). It accounts for approximately half of deaths in dogs over the age of ten and approximately one in four dogs will develop cancer during their lifetime (Adams et al. 2010).

As in human patients, animals with oncological diseases suffer not only from a localised lesion but also from various problems resulting from the neoplastic spread, which cause systemic effects contributing to the reduction of the patient’s general condition and quality of life (Stephens 2019). The importance and participation of animals in people’s lives as family members raises concerns for the quality at the end of life of dogs and cats (Bishop et al. 2016).

In view of the foregoing, veterinarians often need to be aware of the guidelines for pain control and maintenance of the quality of life of cancer patients. Hence, the importance of understanding palliative care, which originated in human medicine, and aims to meet the need to alleviate patients’ suffering in cases where there is no possibility of a cure (Bishop et al. 2016).

The purpose of this review is to approach the palliative care that can be used in veterinary medicine in the face of a patient diagnosed with malignant neoplasia in whom there is no longer a chance of a cure and who suffers from the systemic consequences of neoplastic advancement.

## LITERATURE REVIEW

### History and definition

According to the World Health Organization (WHO), palliative care is defined as: “An approach that improves the quality of life of patients and their families facing a life-threatening illness through the prevention and relief of suffering, including treatment of pain and other physical problems, treatment psychosocial and spiritual, understanding death as a natural process, neither hastening nor postponing it. It is applicable at the beginning of the course of the disease, in conjunction with other therapies aimed at prolonging life (...)” (Sanchez-Cardenas et al. 2022).

The first citations involving palliative care occurred in the early Christian era, in hospices (inns), places that housed pilgrims and travellers, in addition to receiving sick, dying, hungry people, the poor, and orphans (Marocchino 2011).

The term “*animal hospice*” began in the United States in the 1980s, when a small group of veterinarians and health professionals, who already understood *hospice* care in humans, came together to formulate the concept of care and comfort for terminally ill companion animals (Marocchino 2011; Dickinson and Hoffmann 2019).

In the early years, despite several attempts to convince their colleagues that a pet *hospice* was a viable option, pioneers Cloughs, Hancock, Harris, and Mader were unsuccessful, as few were ready to follow in their footsteps (Clark 2000). Subsequently, they began to present data on their experiences on small animals at various congresses and conferences (Marocchino 2011).

Since then, the concept of *hospice* and palliative care spread rapidly with the main objective of maintaining the quality of life.

In 1996, The Nikki Hospice Foundation for Pets (NHFP) was created; the first non-profit organisation whose target was animals with terminal diseases; and it was only in 2009 when The International Association of Animal Hospice and Palliative Care (IAAHPC) was created in the United States, which publishes, every six months, updates on palliative care in veterinary medicine (Marocchino 2011; Bishop et al. 2016).

The terms palliative care and *animal hospice* are often confused, despite having different definitions. Palliative care aims to promote the quality of life for patients with advanced clinical diseases in which there are no more conditions for a cure. In addition to relieving pain and other physical injuries, it provides emotional support to owners, always considering the death as a natural flow. The term *hospice*, on the other hand, is broader than palliative care, involving patients at the end of life (Goldberg 2016) and has an interdisciplinary team that accompanies both the patient and the owners, involving the veterinarian, psychologists, crematoriums/cemeteries, and other necessary professionals (Bishop et al. 2016).

## CANCER IN ANIMALS

Cancer has become a major health concern for domestic animals, given the high occurrence of neoplasms in dogs and cats. It is responsible for about half of the causes of death in small animals over 10 years of age and approximately one in four dogs will develop some type of neoplasm during their lifetime (Adams et al. 2010). In cats, neoplasms, along with infectious diseases and kidney failure, are the main causes of serious illness and death (Fan 2014).

In addition to being responsible for the high mortality in companion animals, cancer can cause pain and suffering in terminally ill patients, contributing to a reduction in the quality of life (Fan 2014). Paraneoplastic syndromes, systemic changes induced by tumours, contribute to the high morbidity of cancer, reducing the general condition of the patient and contributing to a decrease in the quality of life of these animals. Cachexia is an example of a common neoplastic syndrome in veterinary oncology (Fan 2014; Simon and Steagall 2017).

Advances in veterinary health, combined with the development of advanced treatment centres, enable the adoption of more effective therapeutic techniques for the treatment of cancer in companion animals. The development of the palliative care area presupposes the recognition that each patient can be helped, regardless of the evolution of the disease, using supportive therapies, whose objective is to maintain the quality of life of the animals (Simon and Steagall 2017).

## ASSESSMENT OF THE QUALITY OF LIFE OF CANCER PATIENTS IN PALLIATIVE CARE

When establishing the diagnosis of a malignant neoplasm in a pet, it is appropriate to create dialogue with the owner to discuss, in advance, the entire process of the evolution of the disease until the end of the patient’s life. Although it is essential to prioritise the quality of life of the animal (Mason 2016), it must be considered that the quantification of such quality of life is subjective and must be analysed in a particular way, since each animal is unique (Downing 2020). It is also important to assess the emotional profile and financial resources of the family to care for the animal that is approaching death.

From then on, a quality-of-life scale was developed to assist veterinarians and owners in verifying the effectiveness of palliative care, offering an objective assessment of what is very subjective (Villalobos 2011; Downing 2020). In this context, the following parameters are evaluated: pain, hunger, hydration, hygiene, happiness, mobility, and more good days than bad days (Table 1). Each item evaluated is scored from zero to ten and a sum equal to or greater than 35 of these topics suggests an acceptable quality of life indicating that the animal is able to integrate or continue palliative treatment (Villalobos 2011).

**Table 1:** Quality of life scale adapted from Villalobos (2011)

Score	Evaluated criteria
0–10	**PAIN** – Correct pain control and the animal’s ability to breathe is the primary concern. Is the animal’s pain properly controlled? Can the animal breathe normally? Is the use of oxygen necessary?
0–10	**HUNGER** – Does the animal feed sufficiently? Is hand assistance necessary for the animal to accept food better? Is the use of a probe indicated?
0–10	**HYDRATION** – Is the animal hydrated or is there dehydration observed? For patients who do not drink water properly, it is necessary to use subcutaneous fluid therapy on a daily basis.
0–10	**HYGIENE** – Is the animal capable of fulfilling its physiological needs far from where it is located? Do you keep the cleanliness independent? Does the disease interfere with the patient’s hygiene?
0–10	**HAPPINESS** – Does the animal show enthusiasm and interest? Is the animal attentive and does it relate to the family, toys, etc.? Is it a depressed, lonely, anxious, bored, or fearful animal? Can you move the pet bed close to family activities?
0–10	**MOBILITY** – Can the animal stand up without assistance? Do you need any human or mechanical help? Does the animal want to go for a walk? Does the patient constantly have seizures or imbalance?
0–10	**MORE GOOD DAYS THAN BAD DAYS** – When bad days outweigh good days, the quality of life can be greatly compromised.
	
Total	Greater than or equal to 35 represents an acceptable quality of life.

Source: Villalobos (2011)

This scale provides useful guidelines that allow the team of professionals, together with the owners, to discuss the evolution of the disease, since it provides data that allow everyone to evaluate the aspects of the patient’s well-being. The quality of life scale, with its objective score, helps family members face reality in the difficult decision-making process of keeping the pet or choosing the euthanasia option (Villalobos 2011).

## PALLIATIVE MEDICAL CARE IN VETERINARY ONCOLOGY

### Pain management

Although the prevalence of cancer pain in animals is unknown, it is believed to be underestimated and often underdiagnosed (Mason 2016; Elliott and Alderson 2019). Based on epidemiological studies in humans, pain is present in 30% of patients at the initial diagnosis of the disease, and 65% to 85% will show signs of pain during disease progression (Foley 2005).

In research conducted with veterinarians, it was observed that 87% of professionals agree that pain caused by cancer is underdiagnosed and 66% disagree with the statement that cancer pain is easily treated (Bell et al. 2014).

Since, at the time of diagnosis, dogs or cats often already have advanced cancer, it is prudent to infer that a large percentage of veterinary patients have suffered from pain due to disease progression before any medical intervention (Fan 2014; Simpson et al. 2017). However, as many patients have no obvious clinical signs of pain, therapeutic management can be challenging (Mason 2016).

The origin of cancer pain may be related to the primary tumour and metastatic spread (Martins 2015; Elliott and Alderson 2019) and may result from the direct invasion of neoplastic cells into the tissues, visceral stretching, and organ distension or obstruction (Martins 2015; Elliott and Alderson 2019). Neuropathic pain may be a consequence of local nerve compression by neoplastic cells, neuroma formation after surgical resection of the primary tumour, fibrosis, neuritis, and sequelae of specific chemotherapeutic agents (Fan 2014). Data published by Antunes et al. (2008) allow us to conclude that, in more than 60% of dogs and cats diagnosed with cancer, pain occurs due to metastasis, tumour bone infiltration, nerve compression, or excessive growth in the nodule compressing adjacent tissues.

For cancer pain management to be performed optimally, health professionals must understand the pathophysiology of pain, the different types of pain, and, above all, have the know-how to recognise it in pets (Fan 2014; Elliott and Alderson 2019).

For owners, it may be difficult to recognise pain and discomfort in their pets, but veterinary professionals who care for cancer patients should be attentive to the diagnosis and relief of pain caused by the neoplastic process (Dibernardi 2020). Such recognition is fundamental for cancer care that will promote the animal’s quality of life (Fan 2014; Dibernardi 2020).

Owners play a fundamental role in recognising the pain of their pets, as they are attentive to behavioural changes that may represent pain or discomfort (Bennet and Morton 2009), such as changes in the animal’s posture and movement, selfcare, lack of appetite, focal licks, salivation, vocalisation, alteration in breathing, and in defecation and urination patterns (Fan 2014).

The World Health Organization (WHO) has established some guidelines for the treatment of pain that can be extrapolated to the management of dogs and cats with neoplasms (Sanchez-Cardenas et al. 2022), which is a three-step analgesic ladder to control mild, moderate, and severe pain (Thapa et al. 2011). Based on these recommendations as a guidance tool, non-steroidal anti-inflammatory drugs (NSAIDs) are indicated for the treatment of mild pain; for the control of moderate pain, the addition of opioids such as codeine or tramadol is indicated. If pain is not controlled with this combination, the use of more potent opioids is indicated (Fan 2014; Simon and Steagall 2017). The analgesic therapy can be supplemented with anticonvulsants, such as gabapentin, and tricyclic antidepressants, such as amitriptyline, in cancer patients with chronic pain (Fan 2014).

NSAIDs are indicated for the control of nociceptive pain in companion animals, whose mechanism of action is the inhibition of cyclooxygenase (COX) enzymes. In the case of cancer pain, COX-2 is the selective target of inhibition, due to its function in inflammatory pain, which is generated by the production of prostaglandin E_2_. Prostaglandins play an important role in peripheral sensitisation triggering a state of hyperalgesia (Fan 2014). NSAIDs are an important tool in the palliative setting with regard to their analgesic and antineoplastic effects since many tumour cells express receptors for COX-2 (Dore 2011; Elliott and Alderson 2019) and, in addition, many tumours have inflammatory cells in their microenvironment which benefit from anti-inflammatory therapy (Queiroga et al. 2010; Dore 2011). NSAIDs have antiangiogenic activity, which is why they are often used in metronomic chemotherapy protocols to slow neoplastic growth (Elliott and Alderson 2019).

There is evidence that tramadol hydrochloride does not provide sufficient analgesia for treating chronic pain in dogs (Budsberg et al. 2018), although Guedes et al. (2018) reported improvement in the quality of life in cats with chronic pain that used tramadol; this may be due to differences in the metabolism between these species (Elliott and Alderson 2019). Even so, some animals seem to benefit from its use, and we should consider it in a multimodal analgesia (Elliott and Alderson 2019). Flor et al. (2013) demonstrated that the association of tramadol with metamizole, with or without NSAIDs, was effective in the management of moderate to severe cancer pain in dogs with improved quality of life scores.

Anticonvulsants are useful adjuvant analgesics in patients with neuropathic pain, as well as in chronic pain with central sensitisation (Fan 2014; Kremer et al. 2016). Thus, gabapentin, a structural analogue of gamma-aminobutyric acid that acts on calcium channels of the presynaptic terminal reducing the release of neurotransmitters, is indicated in dogs and cats with chronic pain of neuropathic origin (Cashmore et al. 2009; Elliott and Alderson 2019). Its main side effect is sedation and, therefore, its dose must be adjusted to reduce it, especially at the beginning of therapy (Elliott and Alderson 2019). According to Grubb (2010), the administration of gabapentin should not be abruptly interrupted, as it can induce seizure episodes, even in patients without a history.

The use of corticosteroids may be indicated to relieve cancer pain symptoms, specifically when there is a strong inflammatory component. In addition, these drugs can cause a general euphoria effect, which contributes to an improvement in the quality of life in palliative patients. Patients with round cell tumours (lymphoma, mastocytoma, and histiocytic sarcoma) may benefit from corticosteroid therapy, compared to the use of NSAIDs, due to their potential for antitumor activity in these neoplasms (Elliott and Alderson 2019).

Some tricyclic antidepressants, such as amitriptyline, can be used as first-line co-analgesics in chronic cancer pain of neuropathic origin (Kremer et al. 2016; Elliott and Alderson 2019). Its analgesic action is due to the inhibition of serotonin and norepinephrine reuptake, which increases the antagonism of endogenous opioids. The dose indicated for the analgesia is lower than that required for its antidepressant action and may present seizures and arrhythmias as the side effects (Fan 2014; Elliott and Alderson 2019).

It is worth mentioning the importance of cannabinoids in analgesic therapy, especially in chronic pain triggered by neoplasms (Elliott and Alderson 2019; Repetti et al. 2019). Despite the scarce literature data on the use of cannabinoids in the treatment of cancer pain in dogs, Repetti et al. (2019) believe that such patients can benefit from this therapeutic modality, providing an improvement in the quality of life of animals.

For Fan (2014), maintaining the quality of life of animals in cancer therapy should be a priority, based on the disease evolution, in which pain becomes more severe and frequent. Therefore, analgesic protocols should be instituted in an early and rational manner.

### Metronomic chemotherapy

Metronomic chemotherapy consists of the administration of cytotoxic drugs at doses lower than those used in conventional chemotherapy, preferably administered orally, providing low and continuous circulating levels of these drugs (Rodigheri and DeNardi 2013; Barros and Repetti 2015; Diber-nardi 2020).

Its use guarantees cytotoxic, antiangiogenic effects that modify tumour immunology, with low rates of side effects (Barros and Repetti 2015; Dibernardi 2020). Its advantages are its low toxicity, ease of administration, low cost, and lower rates of resistance to antineoplastic drugs (Rodigheri and DeNardi 2013).

The main indications are the palliative control of recurrent, inoperable, or metastatic neoplasms and the treatment of patients who are extremely debilitated or whose owners reject conventional therapies due to the risks of adverse effects (Barros and Repetti 2015).

### Nutritional support

As in human patients, cancer and its common clinical manifestation of cachexia are undesirable in domestic animals. Nutritional support becomes important in palliative care because it can help slow the progression of the inevitable changes in the appetite and body condition of the cancer patient (Saker 2021).

One of the causes of malnutrition that occurs in neoplasms may be the reduction in feed intake. Anorexia, often related to low nutrient intake, change in taste, loss of appetite, and malaise (Saker 2021), can also occur due to the side effects induced by the established therapy (Garcia et al. 2009; Saker 2021). Dysphagia that occurs in upper gastrointestinal tract neoplasms also contributes to anorexia, as do gastrointestinal motility disorders that result in nausea, vomiting, regurgitation (Ockenga and Valentini 2005).

Another important aspect of cancer malnutrition involves the release of inflammatory mediators, including tumour necrosis factor, interleukin (IL) -1, and IL-6 that control appetite and nutrient utilisation by the body. This is the main cause of cancer cachexia. Cytokines produced by tumour proliferation are responsible for the degradation of the lean muscle mass, for increasing the proteolysis and accelerating the cachectic state (Cederholm and Morley 2016).

Cachexia is a complex, multifactorial syndrome characterised by the widespread consumption of body, muscle, and adipose tissues, with progressive weight loss, anaemia, negative nitrogen balance, immune dysfunction, and metabolic changes generally associated with anorexia (Silva 2006). Its incidence in dogs is around 25% in the most varied neoplasms and occurs due to changes in the metabolism of carbohydrates, proteins, and lipids (Bergman 2013).

The occurrence of cachexia associated with cancer significantly affects the patient’s quality of life, providing difficulty in supporting treatment, a reason that highlights the importance of early nutritional intervention in the treatment of dogs and cats with cancer (Case et al. 2011).

In view of this, nutritional therapy becomes fundamental for the treatment of neoplastic cachexia in which adequate diets or medications are indicated to stimulate appetite in these animals. The diet should be highly palatable, provided not very hot to the animal and in a quiet place. Medications that stimulate appetite are also indicated, as are those to control nausea and vomiting (Garcia et al. 2009).

In more advanced cases, in which the hungry animal is unable to ingest food, it is necessary to use enteral tubes (Shearer 2011).

## BIOETHICAL CONSIDERATIONS IN PALLIATIVE TREATMENT

When the patient no longer responds to the treatment options available for palliative care and approaches the end of life, the dialogue with the owner must be guided by the four principles of medical bioethics, namely respect for autonomy, non-maleficence, beneficence, and justice (Beauchamp and Childress 2019).

Respect for autonomy is the obligation of the veterinarian to provide the necessary information about the results of the treatment of the animal, in a clear and complete manner, so that the owner makes the best decision. The team must respect the owner’s beliefs, privacy, and confidentiality, not perform any therapeutic procedure without obtaining the owner’s consent, and must also consider the patient’s autonomy, respecting their preferences in receiving care (Bishop et al. 2016; Downing 2020).

Non-maleficence is the bioethical principle of “causing no harm”, avoiding pain and discomfort for the patient as a result of clinical intervention, as well as avoiding insensitive conduct, on the part of the veterinary team, which may aggravate the owner’s grief experience. Beneficence, on the other hand, includes honest conversations with the owner about the prognosis of the animal and the costs involved, in addition to the discussion about the possibility of euthanasia in those cases in which the continuation of the treatment will result in greater suffering to the patient (Bishop et al. 2016). The last bioethical principle is justice, in which the professional must provide all owners with their best effort in the name of animal welfare (Bishop et al. 2016; Downing 2020).

Often, for religious or cultural reasons, some owners prefer natural death to assisted death (Yeates and Main 2010; Downing 2020). Faced with this situation, it is difficult for the medical team to compromise their professional ethics to justify the treatment of a terminally ill patient who will suffer with pain and other consequences of the evolution of the disease (Yeates and Main 2010).

The ideal would be for an animal to die at home, in a calm and painless state, which could occur in those cases where there is supervision by the veterinary team to provide relief from the symptoms of the disease worsening. However, not all patients receive professional help or die peacefully at home. It is an unpleasant experience to witness the traumatic death of a pet when there is no euthanasia option (Villalobos 2011).

## THE EUTHANASIA DECISION

Handling a terminally ill pet can negatively affect the psychological well-being of human caregivers (Shanan 2015). In view of this, veterinarians must guide them to decision-making options in addressing delicate issues such as: what is the quality of life of the animal? Should the animal undergo palliative care, palliative natural death, or euthanasia? If euthanasia is the option of choice, how should it happen? (Dickinson and Hoffmann 2019)

It should be borne in mind that cancer patients will rarely die as a result of their disease since neoplasm-related morbidity usually leads to euthanasia before natural death occurs (Mason 2016).

When death becomes imminent, it is essential to provide painkillers that also provide sedation to alleviate any suffering (Downing 2020). However, when all options for maintaining the patient’s quality of life are exhausted, interfering with animal welfare, it is acceptable to have euthanasia as an option (Shearer 2011; Villalobos 2011; Bishop et al. 2016).

Euthanasia is a legal procedure for the purpose of alleviating the suffering of an animal, ending life, in a peaceful and humane manner, when other efforts to alleviate distress have failed or cannot be pursued (Bishop et al. 2016).

Considering that animal comfort should always be prioritised during euthanasia, the domestic environment will often be the appropriate place to perform such a procedure (Bishop et al. 2016) since there will be no need to move the debilitated animal, often in pain, to be transported to a clinic, in addition to offering greater privacy to the owners. If the procedure needs to be performed in a clinic, the owners must remain with the animal at all times (Cooney et al. 2012).

For the proper euthanasia procedure, it is recommended that pre-euthanasia sedation or anaesthesia be performed whenever possible in order to reduce the environmental stressors.

## FINAL CONSIDERATIONS

Over the years, dogs and cats have achieved increased longevity and are frequently diagnosed with neoplasms. In order to maintain the quality of life of these animals, especially when the treatment of the primary disease does not present satisfactory results and the signs of the disease spread, there is a need for the institution of palliative care. At this stage, pain and other symptom control measures are taken to maintain the animal welfare, reflecting the desire to ensure that non-human family members are ethically cared for at the end of their lives. When palliative care is performed, the option of euthanasia is not completely ruled out, but it is not the only alternative in cases of a terminal diagnosis.
